# Linezolid brain penetration in neurointensive care patients

**DOI:** 10.1093/jac/dkae025

**Published:** 2024-02-07

**Authors:** Arthur Hosmann, Miriam M Moser, Wisse van Os, Leon Gramms, Valentin al Jalali, Maria Sanz Codina, Walter Plöchl, Constantin Lier, Frieder Kees, Christoph Dorn, Karl Rössler, Andrea Reinprecht, Markus Zeitlinger

**Affiliations:** Department of Neurosurgery, Medical University of Vienna, Vienna, Austria; Department of Neurosurgery, Medical University of Vienna, Vienna, Austria; Department of Clinical Pharmacology, Medical University of Vienna, Vienna, Austria; Department of Clinical Pharmacology, Medical University of Vienna, Vienna, Austria; Department of Neurosurgery, Medical University of Vienna, Vienna, Austria; Department of Clinical Pharmacology, Medical University of Vienna, Vienna, Austria; Department of Clinical Pharmacology, Medical University of Vienna, Vienna, Austria; Department of Anesthesia, General Intensive Care Medicine and Pain Management, Medical University of Vienna, Vienna, Austria; Institute of Pharmacy, University of Regensburg, Regensburg, Germany; Department of Pharmacology, University of Regensburg, Regensburg, Germany; Institute of Pharmacy, University of Regensburg, Regensburg, Germany; Department of Neurosurgery, Medical University of Vienna, Vienna, Austria; Department of Neurosurgery, Medical University of Vienna, Vienna, Austria; Department of Clinical Pharmacology, Medical University of Vienna, Vienna, Austria

## Abstract

**Background:**

Linezolid exposure in critically ill patients is associated with high inter-individual variability, potentially resulting in subtherapeutic antibiotic exposure. Linezolid exhibits good penetration into the CSF, but its penetration into cerebral interstitial fluid (ISF) is unknown.

**Objectives:**

To determine linezolid penetration into CSF and cerebral ISF of neurointensive care patients.

**Patients and methods:**

Five neurocritical care patients received 600 mg of linezolid IV twice daily for treatment of extracerebral infections. At steady state, blood and CSF samples were collected from arterial and ventricular catheters, and microdialysate was obtained from a cerebral intraparenchymal probe.

**Results:**

The median *f*AUC_0–24_ was 57.6 (24.9–365) mg·h/L in plasma, 64.1 (43.5–306.1) mg·h/L in CSF, and 27.0 (10.7–217.6) mg·h/L in cerebral ISF. The median penetration ratio (*f*AUC_brain_or_CSF_/*f*AUC_plasma_) was 0.5 (0.25–0.81) for cerebral ISF and 0.92 (0.79–1) for CSF. Cerebral ISF concentrations correlated well with plasma (R = 0.93, *P* < 0.001) and CSF levels (R = 0.93, *P* < 0.001).

The median *f*AUC_0–24_/MIC ratio was ≥100 in plasma and CSF for MICs of ≤0.5 mg/L, and in cerebral ISF for MICs of ≤0.25 mg/L. The median *fT*_>MIC_ was ≥80% of the dosing interval in CSF for MICs of ≤0.5 mg/L, and in plasma and cerebral ISF for MICs of ≤0.25 mg/L.

**Conclusions:**

Linezolid demonstrates a high degree of cerebral penetration, and brain concentrations correlate well with plasma and CSF levels. However, substantial variability in plasma levels, and thus cerebral concentrations, may result in subtherapeutic tissue concentrations in critically ill patients with standard dosing, necessitating therapeutic drug monitoring.

## Introduction

Linezolid is indicated for the treatment of drug-resistant severe nosocomial or community-acquired pneumonia and skin and soft-tissue infections caused by Gram-positive bacteria.^1^ Its ability to effectively penetrate into the CSF makes it a valuable salvage therapy for CNS infections.^2–12^ However, there is significant inter- and intra-individual variability in linezolid concentrations, particularly in critically ill patients, potentially resulting in subtherapeutic drug exposure.^2–4,6,7,10,12^ Inadequate antibiotic exposure can have detrimental effects on clinical outcome and may also promote antimicrobial resistance. Currently, there are no reliable data on linezolid brain penetration, and CSF levels have been used as a proxy for cerebral penetration instead.

Microdialysis is a technique to measure the unbound, pharmacologically active drug concentrations *in vivo* beyond the blood–brain barrier.^13–15^ Using this technique, linezolid has already been measured in humans in subcutaneous tissue,^2,16–21^ muscle,^2,16,17,22^ synovial fluid^22^ and cancellous bone.^23^ However, to the best of our knowledge, there are currently no available data regarding the levels of linezolid in the cerebral interstitial fluid (ISF) in either humans or animal models.

The objective of the present study was to determine unbound linezolid concentrations in plasma, CSF and, using cerebral microdialysis, cerebral ISF of neurointensive care patients at steady-state conditions.

## Patients and methods

### Population

Between April 2019 and August 2022, five patients were prospectively included in the study, receiving IV treatment with linezolid while being monitored with cerebral microdialysis. The study was conducted at the Neurosurgical ICU of the Medical University of Vienna and was approved by the local ethics committee (EK1031/2015; EudraCT 2015-000121-37). Patients who met the inclusion criteria were sedated and mechanically ventilated, rendering them unable to provide written informed consent at the time of study inclusion. However, retrospective permission was obtained from the patients once they regained consciousness.

### Multimodality neuromonitoring and cerebral microdialysis

For multimodality neuromonitoring, a NEUROVENT-PTO 2L catheter (RAUMEDIC AG, Helmbrechts, Germany) was implanted side-by-side with a 70 MD Bolt Microdialysis Catheter (M Dialysis AB, Stockholm, Sweden) through a two-lumen bolt system (BOLT KIT PTO 2L, RAUMEDIC AG, Helmbrechts, Germany) into the white matter of the frontal lobe. Probes were placed 1–2 cm anterior to Kocher’s point within the presumed watershed of the anterior cerebral artery and middle cerebral artery, ipsilateral to the ruptured aneurysm. In cases of anterior communicating artery involvement, probes were placed on the side with the maximal extension of the subarachnoid blood clot. Probe locations were verified on CT scan 1 day after the procedure. The tip of the microdialysis probe was positioned at a median depth of 32 mm (IQR: 31–33 mm) beyond the dura. The microdialysis catheter had a 10 mm membrane length with a molecular mass cut-off of 20,000 daltons and was perfused at a flow rate of 0.3 µL/min with ‘Perfusion Fluid CNS’ (M Dialysis AB, Stockholm, Sweden) using a microinfusion pump (107 Microdialysis Pump, M Dialysis AB, Stockholm, Sweden). Cerebral metabolism and linezolid concentrations were determined by collecting microdialysate in microvials (M Dialysis AB, Stockholm, Sweden) every hour. For cerebral metabolism measurement, the microdialysate was then analysed at the bedside using a microdialysis analyser (ISCUSflex, M Dialysis AB, Stockholm, Sweden) to measure cerebral glucose, lactate, pyruvate, glycerol and glutamate concentrations.

An external ventricular drainage was placed through a burr hole at Kocher’s point into the frontal horn of the lateral ventricle for continuous CSF drainage.

After probe implantation a CT scan was performed to verify accurate catheter position and rule out perifocal haemorrhage or oedema. Microdialysis catheter depth was measured on native CT scans from the dura to the catheter’s gold tip in coronal planes.

### Study medication

Each patient received IV administrations of 600 mg of linezolid every 12 h. Linezolid was infused continuously over 60 min through a central venous catheter using a perfusion pump. To achieve steady-state conditions, at least three administrations were considered necessary.

### Sampling and probe handling

Microdialysate was collected 1 h before the start of linezolid infusion to establish baseline concentrations. Subsequently, microdialysate was collected at the second, fourth, sixth, eighth, tenth and twelfth hour after the start of linezolid infusion, immediately placed on ice, and then stored at −65°C. The microdialysate in between these specific timepoints was utilized for routine bedside analysis of cerebral metabolism.

CSF was collected via an external ventricular drainage catheter. The initial millilitre was discarded due to the dead space of the ventricular catheter, and the second millilitre was used for linezolid pharmacokinetics (PK) analysis. CSF sampling was conducted just before the initiation of linezolid infusion, after 1 h, 2 h, and then every 2 h throughout the 12 h dosing interval. Simultaneously with CSF sampling, plasma samples were obtained from an arterial catheter. Both blood and CSF samples were immediately centrifuged at 4°C, 2500 **g** for 10 min, and the supernatant was collected and stored below −65°C.

The creatinine clearance was calculated using the Cockcroft and Gault equation: creatinine clearance = [(140 − age in years) × (weight in kg) × (0.85 if female)]/[72 × (serum creatinine in mg/dL)].

### Retrodialysis

Retrodialysis was conducted to determine the individual *in vivo* probe recovery for linezolid. The cerebral microdialysis catheter *in situ* was perfused with a solution containing 20 mg/L linezolid (*C*_in_) at a flow rate of 0.3 µL/min for each patient. After an equilibration period of 90 min, two consecutive microdialysis samples with a collection interval of 1 h each were collected and the average linezolid concentration (*C*_out_) was calculated. To determine recovery by loss, the individual relative recovery was calculated as the mean ratio of drug lost during passage (*C*_in−_*C*_out_) and drug entering the microdialysis probe (*C*_in_).

The linezolid concentration in each microdialysis sample was adjusted for the individual *in vivo* probe recovery. Therefore, the absolute cerebral ISF was calculated for each sample as follows: 100 × (sample concentration/relative recovery).

### Drug assay

Linezolid concentrations were determined by HPLC-UV using a Prominence LC20 modular HPLC system equipped with an SPD-M30A PDA detector (set to 254 nm) and LabSolutions software (Shimadzu, Duisburg, Germany). Separation was performed using a CORTECS T3 2.7 μ 100 × 3 mm analytical column (Waters, Eschborn, Germany) preceded by a guard column (NUCLEOSHELL RP18 2.7 μ 4 × 3 mm column protection system, Macherey-Nagel, Düren, Germany). The mobile phase consisted of 0.1 M sodium phosphate buffer/acetonitrile 79:21 (v/v), with final pH 6.5. At a flow rate of 0.4 mL/min, linezolid eluted after 3.7 min. The total concentrations of linezolid in plasma or CSF were determined after deproteinization of 100 µL of plasma or CSF with 100 μL of 7% perchloric acid; the free plasma concentrations (*C*_free_) were determined after ultrafiltration of 300 µL of plasma buffered with 10 µL of 3 M potassium phosphate, pH 7.4, using Vivafree^TM^ 500 30 kDa Hydrosart^®^ centrifugal ultrafiltration devices (Vivaproducts Inc., Littleton, MA, USA) as described previously.^24,25^ Microdialysate was injected directly. Injection volume was 1 µL for all samples. The linearity in plasma or saline (as surrogate for CSF or ultrafiltrate) has been proven from 0.1 to 30 mg/L (R > 0.9991). The lower limit of quantification (signal:noise 6:1) was 0.05 mg/L in plasma, and 0.03 mg/L in plasma ultrafiltrate or CSF, respectively. Based on in-process quality control (QC) samples, the intra- and inter-assay precision in plasma or saline (coefficient of variation, CV) was <8%, the relative error in accuracy was <3%. Regarding free linezolid plasma concentrations, the accuracy cannot be specified as the protein binding in an individual plasma sample and accordingly the true free concentration is unknown.^26^ The unbound fraction (*f_u_* = *C*_free_/*C*_total_ × 100%) in spiked pooled plasma from healthy subjects was 88.1% ± 5.5% (CV 6.3%). *C*_free_ was determined in three plasma samples of each patient and the individual mean *f_u_* was calculated and used for the calculation of the individual free plasma PK profiles.

### Pharmacokinetics/pharmacodynamics (PK/PD)

Non-compartmental PK analysis was performed using Phoenix WinNonlin (version 8.3; Certara, Princeton, NJ, USA). The maximum concentration (*C*_max_), time to maximum concentration (*T*_max_), terminal elimination half-life (*t*_½_) and the area under the concentration–time curve for free drug from 0 to 12 h (*f*AUC_0–12_) were additionally determined. For plasma, the volume of distribution (*V*_d_) and clearance (CL) were calculated. *f*AUC_0–12_ was multiplied by two to obtain *f*AUC_0–24_. The ratio between the *f*AUC in cerebral ISF or CSF and plasma (*f*AUC_brain_or_CSF_/*f*AUC_plasma_) was calculated as a measure of linezolid penetration. Microdialysis observations were assigned to the midpoint of the collection interval for PK analysis. For one patient, insufficient data were available to perform CSF PK analysis.

The pharmacokinetic/pharmacodynamic (PK/PD) indices that correlate best with linezolid activity are the percentage of the dosing interval during which unbound drug concentrations exceed the MIC of a pathogen (*fT*_>MIC_) and the *f*AUC/MIC ratio.^1,27^ Since linezolid PK/PD targets for efficacy in CNS infections are not available, an *fT*_>MIC_ of ≥80% and an *f*AUC_0–24_/MIC of ≥100 were used as PK/PD targets, as shown to be related to clinical success in the treatment of bacteraemia, lower respiratory tract infection, and skin and skin-structure infections.^27–29^ The *fT*_>MIC_ was determined by estimating the time of intersection of the PK profile and the respective MIC value using Phoenix WinNonlin. For two patients, PK data were collected only until 8 h after dosing. To calculate *f*AUC_0–12_ and *fT*_>MIC_ for these patients, PK profiles were extrapolated until 12 h after dosing using the individual terminal elimination rates determined in the PK analysis.

### Statistics

Statistical analysis was conducted using SPSS^®^ Statistics 22 (IBM Corp., Armonk, NY, USA) and MS Excel 2011 for Mac (Microsoft, Redmond, CA, USA). Results are presented as median and range. Correlations were calculated using the Spearman correlation coefficient. Statistical significance was evaluated using a two-sided significance level with a threshold set at *P* < 0.05.

## Results

### Population

This prospective study included five patients (three female, two male) with a median age of 53 years (range 25–61 years) and a median BMI of 26.2 kg/m^2^ (range 23.4–29.4 kg/m^2^).

All patients suffered from aneurysmal subarachnoid haemorrhage (SAH), with a median Hunt & Hess grade of 4 (range 3–5) at admission. In four cases the aneurysm was clipped, and in one patient it was coiled within 48 h after the haemorrhage. The microdialysis probe was implanted at a median of 3 days (range 1–3 days) following SAH and was positioned into the frontal lobe (three right, two left) at a depth of 30–33 mm. No ischaemia or bleeding at the implantation site were observed on CT scans.

Linezolid was administered in four patients due to severe pneumonia and in one patient due to sepsis. Laboratory results on the day of linezolid sampling are shown in Table 1. Demographics and individual laboratory results of each patient are presented in Table S1 (available as Supplementary data at *JAC* Online). CSF cell count was elevated in all patients, most likely as a reaction to the SAH, as CSF glucose was within the physiological range and in none of the patients was a pathogen detected in CSF cultures.

**Table 1.** Multimodality neuromonitoring parameters and results of laboratory investigations during linezolid sampling

Parameter	Median (range)
Laboratory investigations	
C-reactive protein (mg/dL)	5.2 (1.7–32)
Leucocytes (×10^9^/L)	9.3 (6–27.4)
Creatinine (mg/dL)	0.9 (0.3–1.7)
Creatinine clearance (mL/min)	107.9 (47.4–341.3)
Gamma-glutamyl transpeptidase (U/L)	461 (202–662)
Glutamic pyruvic transaminases (U/L)	95 (39–211)
Glutamic oxaloacetic transaminase (U/L)	90 (37–331)
Lactate dehydrogenase (U/L)	267 (238–517)
Alkaline phosphatase (U/L)	193 (92–409)
Albumin (g/L)	28 (26.1–31.4)
CSF (*n* = 9)	
Cell count (absolute/µL)	188 (10–1196)
Glucose (mg/dL)	59 (48–103)
Lactate (mmol/L)	5.1 (2.1–6.3)
Protein (mg/dL)	102 (29–236)
Multimodality monitoring	
Mean blood pressure head level (mmHg)	86 (75–107)
Intracranial pressure (mmHg)	9.9 (4–12)
Cerebral perfusion pressure (mmHg)	82 (60–82)
Brain tissue oxygen tension (mmHg)	29 (8–39)
Lactate/pyruvate ratio	39 (29–61)

The patients received sedation through continuous infusions: either sufentanil and midazolam (Patients 1, 2 and 5) or propofol and remifentanil (Patients 3 and 4). Deep sedoanalgesia in Patient 1 involved ketamine and propofol infusion. Dexmedetomidine and clonidine were added to the regimen for Patients 3 and 4, respectively. Patient 3 also received oral quetiapine 100 mg every 6 h, and Patient 2 received hydrocortisone infusion.

In addition to linezolid, all patients received meropenem 2 g every 8 h. Patient 2 also received IV anidulafungin 100 mg daily.

Nimodipine was administered either orally (60 mg every 4 h for Patients 1 and 3) or IV (1–2 mg/h for Patients 2, 4 and 5). All patients received oral pantoprazole 40 mg and subcutaneous enoxaparin sodium 40 mg daily. For seizure prophylaxis/treatment, IV levetiracetam (500–1500 mg) was administered to Patients 1, 3, 4 and 5. IV metamizole 1 g every 8 h was given to Patients 1, 3, 4 and 5. Patients 1 and 5 received metoclopramide 10 mg and erythromycin 100 mg every 8 h as a prokinetic agent. Continuous insulin infusion was performed in Patients 2 and 4.

Mean multimodality neuromonitoring parameters during linezolid measurement are presented in Table 1. Throughout the observation period, no elevation of intracranial pressure was observed and brain tissue oxygen tension was within the physiological range, except for one patient who exhibited cerebral hypoxia (7.5 ± 5.2 mmHg). However, cerebral microdialysis in this patient revealed only mild signs of ischaemia, indicated by a modest decrease in cerebral glucose (0.8 ± 0.6 mmol/L) and slightly elevated lactate/pyruvate ratio (37 ± 2), along with normal cerebral lactate (2.1 ± 1.5 mmol/L) and pyruvate (76.8 ± 23.6 µmol/L) levels.

### Sampling characteristics

The PK of linezolid were measured at a median interval of 10 days (range 6–21 days) after SAH and 7 days (range 5–18 days) after microdialysis probe implantation. The samples were taken under presumed steady-state conditions, as a median number of 6 doses (range 5–12 doses) were administered before PK sampling. In two patients, the 8 h plasma, CSF and microdialysis samples are missing due to organizational reasons. In one patient, two microdialysis samples are missing due to probe malfunction (kinking of the outlet tube). Additionally, in one patient with slit ventricles, three out of eight CSF samples could not be drawn.

The median *f_u_* in plasma across all patients was 95.2% (range 91.6%–99.3%).

Retrodialysis was performed in all patients at a median interval of 1 day (range 1–4 days) after linezolid PK sampling. The median relative recovery for linezolid was 66% (range 43%–69%).

### PK/PD

**Figure 1.**
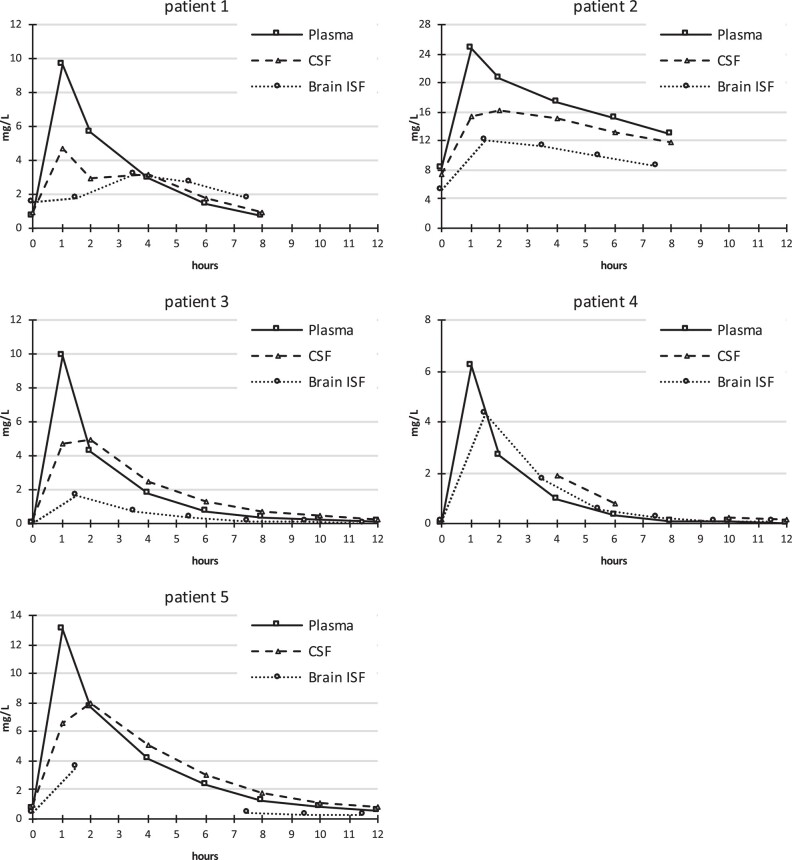
Linezolid PK in plasma, CSF and cerebral ISF in each patient at steady state following twice-daily IV infusions of 600 mg over 1 h.

Median linezolid concentrations in plasma, CSF and cerebral ISF at steady-state conditions are shown in Figure 1. Median PK parameters are shown in Table 2. Individual PK parameters of each patient are provided in Table S2.

**Table 2.** PK parameters of linezolid under steady-state conditions in plasma (*n* = 5), CSF (*n* = 4) and cerebral ISF (*n* = 5)

Parameter	*f*AUC_0–12_ (mg·h/L)	*f*AUC_0–24_ (mg·h/L)	*f*AUC_brain or CSF_/*f*AUC_plasma_	*C*_max_ (mg/L)	*T*_max_ (h)	*t*_½_ (h)
Plasma_free_	28.8 (12.4–182.5)	57.6 (24.9–365.1)	—	9.9 (6.2–24.8)	1 (1–1)	2.5 (1.9–9.9)
CSF	32.0 (21.7–153.1)	64.1 (43.5–306.1)	0.92 (0.79–1)^a^	6.5 (4.7–16.2)	2 (1–2)	3.0 (2.4–10.6)
Brain	13.5 (5.4–108.8)	27.0 (10.7–217.6)	0.5 (0.25–0.81)^a^	3.6 (1.7–12.1)	1.5 (1.5–3.5)	3.3 (1.7–10.1)

Data are shown as median (range).

^a^ *f*AUC_brain or CSF_/*f*AUC_plasma_ was calculated for patients with both CSF and cerebral ISF values only (*n* = 4).

The median penetration ratio for CSF (*f*AUC_CSF_/*f*AUC_plasma_) was 0.92 (range 0.79–1) and for cerebral ISF (*f*AUC_brain_/*f*AUC_plasma_) it was 0.5 (range 0.25–0.81).

In plasma, the median *V*_d_ was 61.3 L (range: 47.2–137.3 L) and the median CL was 20.9 L/h (range: 3.3–48.2 L/h). CL did not show any significant correlation with age (*P* = 0.87), creatinine clearance (*P* = 0.19) or liver function parameters (*P* > 0.05).

**Figure 2.**
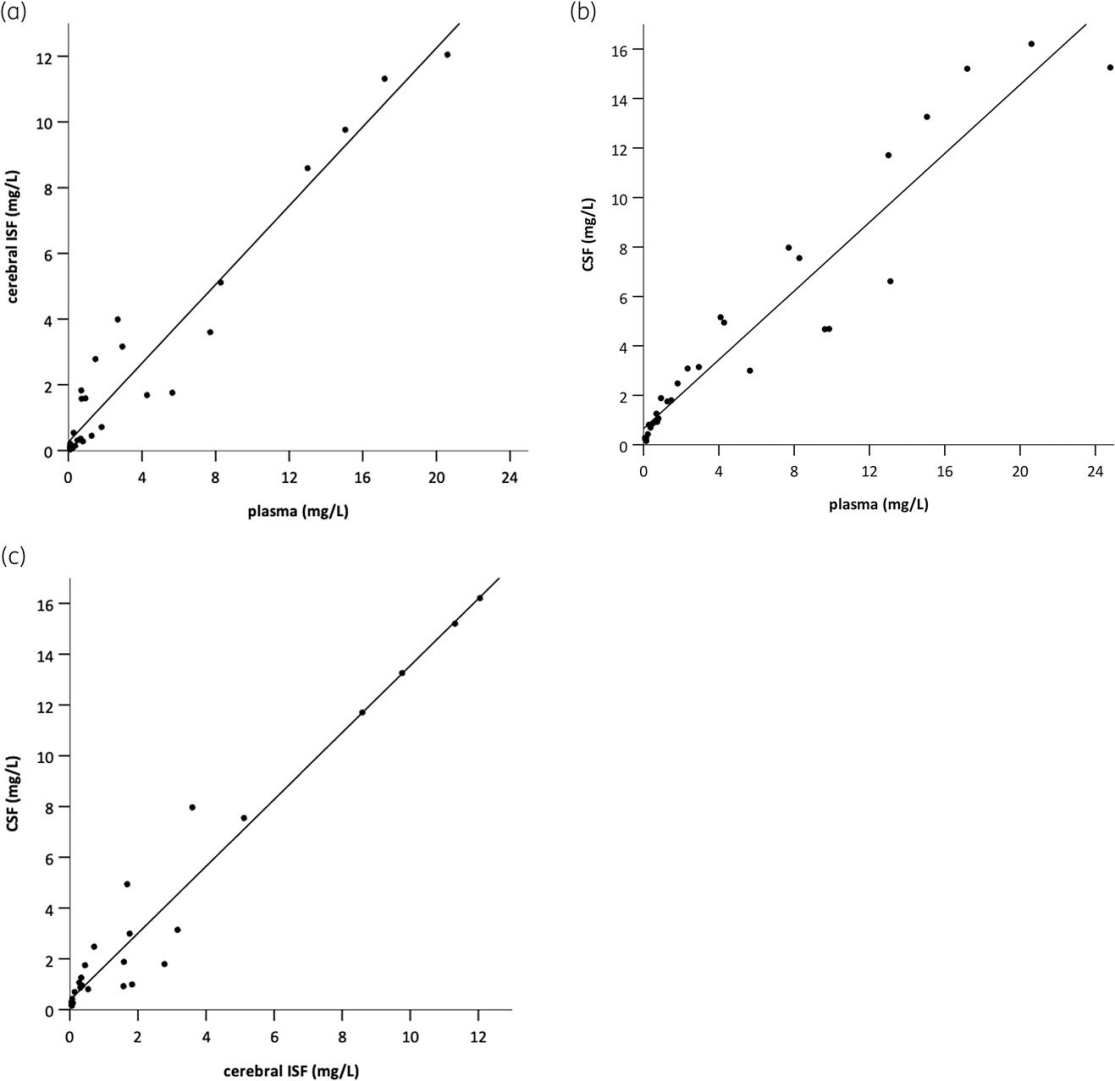
Correlations between free plasma, CSF and cerebral ISF linezolid concentrations at steady state. Plasma concentrations significantly correlated with concentrations in cerebral ISF (R = 0.93, *P* < 0.001) (a) and CSF (R = 0.93, *P* < 0.001) (b). Cerebral ISF concentrations strongly correlated with CSF concentrations (R = 0.97, *P* < 0.001) (c).

Cerebral ISF concentrations of linezolid showed strong correlations with plasma (R = 0.93, *P* < 0.001; Figure 2a) and CSF levels (R = 0.93, *P* < 0.001; Figure 2c). Additionally, linezolid levels in CSF were significantly correlated with plasma concentrations (R = 0.97, *P* < 0.001; Figure 2b).

Table 3 presents the PK/PD parameters at steady state for MICs of 0.125–16 mg/L. The *f*AUC_0–24_/MIC ratios and *fT*_>MIC_ values per patient are provided in Tables S3 and S4, respectively. The median *f*AUC_0–24_/MIC was ≥100 for MICs of ≤0.5 mg/L in plasma (median 115.1; range 49.8–730.1) and CSF (median 128.1; 87–612.3), and for MICs of ≤0.25 mg/L in cerebral ISF (median 107.9; range 42.9–870.5).

**Table 3.** PK/PD index values for a range of MICs at steady state in plasma, CSF and cerebral ISF

		MIC (mg/L)							
		0.125	0.25	0.5	1	2	4	8	16
Plasma	*f*AUC_0–24_/MIC	460.4 (199.1–2920.5)	230.2 (99.5–1460.2)	115.1 (49.8–730.1)	57.6 (24.9–365.1)	28.8 (12.4–182.5)	14.4 (6.2–91.3)	7.2 (3.1–45.6)	3.6 (1.6–22.8)
	*fT*_>MIC_ (h)	12 (7.9–12)	10.9 (6.3–12)	9 (5–12)	7 (3.7–12)	5 (2.2–12)	2.7 (0.9–12)	0.5 (0–12)	0 (0–4.6)
	*fT*_>MIC_ (% of dosage interval)	100 (66–100)	91 (52–100)	75 (42–100)	59 (31–100)	41 (19–100)	22 (7–100)	4 (0–100)	0 (0–38)
CSF	*f*AUC_0–24_/MIC	512.5 (347.9–2449.1)	256.2 (174–1224.5)	128.1 (87–612.3)	64.1 (43.5–306.1)	32 (21.7–153.1)	16 (10.9–76.5)	8 (5.4–38.3)	4 (2.7–19.1)
	*fT*_>MIC_ (h)	12 (12–12)	12 (12–12)	11.2 (9.3–12)	9.3 (6.6–12)	6.3 (4.2–12)	3.1 (0.5–12)	0 (0–11.9)	0 (0–0.6)
	*fT*_>MIC_ (% of dosage interval)	100 (100–100)	100 (100–100)	93 (78–100)	77 (55–100)	53 (35–100)	26 (4–100)	0 (0–100)	0 (0–5)
Brain	*f*AUC_0–24_/MIC	215.9 (85.9–1741.1)	107.9 (42.9–870.5)	54 (21.5–435.3)	27 (10.7–217.6)	13.5 (5.4–108.8)	6.7 (2.7–54.4)	3.4 (1.3–27.2)	1.7 (0.7–13.6)
	*fT*_>MIC_ (h)	11.5 (7.9–11.5)	11.5 (6.0–11.5)	7.1 (4.0–11.5)	4.9 (1.9–11.5)	2.4 (0–11.5)	0 (0–11.5)	0 (0–7.9)	0 (0–0)
	*fT*_>MIC_ (% of dosage interval)	96 (66–96)	96 (50–96)	59 (34–96)	41 (15–96)	20 (0–96)	0 (0–96)	0 (0–66)	0 (0–0)

Data are shown as median (range).

The median *fT*_>MIC_ was ≥80% of the dosage interval for MICs of ≤0.5 mg/L in CSF (93%, range 78%–100%) and for MICs of ≤0.25 mg/L in plasma (91%, range 52%–100%) and cerebral ISF (96%, range 50%–96%).

## Discussion

To the best of our knowledge, this is the first study using cerebral microdialysis for measuring interstitial linezolid concentrations *in vivo* within the human brain.

A previous study investigated total tissue concentrations of linezolid in cerebral biopsies sampled during neurosurgical tumour resection.^3^ In that study, 2 h after IV administration of 600 mg linezolid over 30 min, concentrations in plasma, CSF and brain were reported to be 6.4 ± 2.5, 5.1 ± 3.5 and 2.6 ± 0.8 mg/kg, respectively. These concentrations are considerably lower than the concentrations observed in the present cohort for all three compartments, although the brain concentrations being reported in mg/kg and the shorter infusion duration of 30 min complicates a direct comparison with the present study. Moreover, the brain biopsy samples contained both extracellular and intracellular concentrations of linezolid. Since intracellular linezolid concentrations are 0.5-fold lower than extracellular linezolid concentrations,^30^ the extracellular pharmacologically active linezolid concentrations in the brain, and thus the reported brain penetration of 45%, may have been underestimated.^3^

The precise mechanism underlying linezolid’s brain penetration remains unknown. However, considering the high penetration ratio of linezolid for cerebral ISF and CSF, coupled with its moderate lipophilicity, low molecular weight and low protein binding, passive diffusion emerges as the most likely mode of penetration through the blood–brain barrier.^7,31^ CSF concentrations of linezolid have been investigated in various studies involving neurosurgical patients.^4–7,10–12^ The high penetration of linezolid into CSF in our study, with a median penetration ratio of 92%, is in line with previously published data.^31^ However, other studies reported substantially lower linezolid penetration into CSF, ranging from 25% to 80%,^2–10^ whilst another study reported accumulation of linezolid in CSF, with penetration ratios exceeding 1 in all patients (range 1.2–2.7).^12^ Myrianthefs *et al.*^7^ found much higher linezolid concentrations in CSF despite lower CSF penetration (66%). This can be attributed to higher plasma concentrations and a longer half-life in CSF (19 h) compared with our study, resulting in *fT*_>MIC_ values of 100% for MICs up to 4 mg/L.^7^ In contrast, Luque *et al.*^6^ reported highly variable and generally lower CSF concentrations, even though CSF penetration was 77%. In their large neurosurgical cohort, neither in plasma nor CSF was sufficient linezolid exposure achieved.^6^

Microdialysis studies have indicated that in critically ill patients tissue penetration of linezolid is reduced, and that target site exposure is decreased in morbidly obese subjects.^19^ Additionally, it was shown that renal function can significantly influence linezolid concentrations.^5,32^ In our study population, the patient exhibiting the highest linezolid plasma levels had the lowest creatinine and linezolid plasma clearance. Conversely, patients with higher creatinine and linezolid plasma clearance displayed lower plasma concentrations of linezolid. The substantial variability in linezolid clearance highlights the critical need for dose individualization.

Cerebral infections can likewise exert a significant influence on cerebral linezolid concentrations. Cerebral inflammation can enhance the permeability of the blood–CSF barrier, accompanied by a reduction in CSF production and outflow, leading to drug accumulation in CSF.^33,34^ A previous study in patients with ventriculitis found similar AUC-based penetration of linezolid into CSF but, on average, a longer half-life in CSF compared with the present study, resulting in linezolid concentrations of ≥2 mg/L during almost the entire dosing interval.^11^ In the paediatric population, linezolid exhibited significant penetration into the CSF, demonstrating this ability even in the absence of inflammation. Remarkably, there was no discernible difference in its penetration into the CSF between cases with inflamed meninges and those without.^35^ The location and method of CSF collection may also affect the observed CSF PK. Linezolid concentrations increase along the physiological CSF pathway, with the lowest levels within the lateral ventricles and the highest levels within the subarachnoid space.^36^ Furthermore, removing a ventricular drainage results in increased linezolid concentration within the lumbar subarachnoid space.^36^ These factors may explain the lower CSF concentrations observed in our cohort.

The median *f*_u_ of linezolid in plasma in our study was notably high at >95%. Previous studies have reported an unbound linezolid fraction of 82%–88% in plasma.^2,17,20–22,37^ The high unbound fraction observed in our cohort may be explained by the fact that all patients exhibited hypoalbuminaemia (28 g/L; range 26–31 g/L).

Both *fT*_>MIC_ and *f*AUC_0–24_/MIC are related to the antimicrobial efficacy of linezolid.^27–29^ Looking at the median values obtained in this study, linezolid exposure based on both the *fT*_>MIC_ and *f*AUC_0–24_/MIC targets was insufficient to cover pathogens with MICs of ≥1 mg/L, which is the MIC_90_ for several pathogens commonly causing cerebral infections, such as *Staphylococcus aureus*, *Staphylococcus epidermidis* and *Enterococcus faecalis*.^38^ These findings suggest that dosing linezolid twice daily at 600 mg may be too low to achieve effective drug exposure in CSF and cerebral ISF of neurointensive care patients. This is in line with a study in critically ill patients reporting subtherapeutic plasma concentrations following 600 mg q12h dosing, with high variability between individuals.^39^ Continuous infusions or higher daily doses, potentially with shorter dosing intervals to avoid toxicity, may increase PK/PD target attainment.^40,41^

Overall, the inter-subject variability seems to be driven more by the variability in plasma exposure than by plasma to tissue penetration, indicated by an over 10-fold range for *f*AUC_0–12_ for plasma while the *f*AUC_CSF_/*f*AUC_plasma_ ratio varied only by 20% and the *f*AUC_brain_/*f*AUC_plasma_ ratio varied by approximately 4-fold (Table S2). The large inter-subject variability also results in strong impact on target attainment of up to 4 MIC titre steps (0.125 to 2 mg/L) for the threshold *f*AUC_0–24_/MIC ratios of 100 and *fT*_>MIC_ of 80% (Tables S3 and S4).

Therefore, particularly in critically ill patients, therapeutic drug monitoring of linezolid is recommended to avoid treatment failure.^6,27,32,42–44^ The observed correlations between linezolid concentrations in plasma, CSF and cerebral ISF in this study suggest that plasma concentrations might serve as a potential surrogate marker for target-site exposure.

A significant limitation of this study is the small sample size, stemming from the inherent challenge of encountering a limited number of patients necessitating both cerebral microdialysis and simultaneous linezolid treatment. This limitation not only impacts the statistical power of our findings but also hinders drawing robust conclusions. The very limited sample size of only five patients precluded comprehensive simulations incorporating inter-individual variability. Consequently, the results should be interpreted with caution, recognizing the constraints of our dataset. Despite the prospective study design, missing samples in plasma, CSF and/or brain tissue led to a variable number of samples per patient, limiting the data consistency. This study was conducted in critically ill neurointensive care patients with severe SAH, limiting the generalization of findings to healthy brain tissue, and potential impairment of the blood–brain barrier cannot be ruled out. Caution is advised when extrapolating our results to scenarios involving abscesses, as their presence can alter linezolid PK, causing variations in drug concentrations within the abscess compared with surrounding tissues. Furthermore, the penetration rates and PK profiles were determined exclusively in patients without CNS infection, limiting the generalizability of our findings. Lastly, microdialysis provides focal measurements and cerebral concentrations may vary across distinct brain areas. The positioning of the catheter could impact local perfusion and, consequently, antibiotic delivery.

In conclusion, linezolid showed a high penetration into both CSF and cerebral ISF at steady-state conditions, and strong correlations between concentrations in plasma, CSF and cerebral ISF were observed. However, linezolid exposure in cerebral ISF was typically lower than in plasma and CSF. Relying solely on CSF or plasma concentrations may thus lead to an overestimation of linezolid activity in cerebral ISF. Due to the significant variability of linezolid concentrations observed in all three compartments, therapeutic drug monitoring may be required to ensure effective linezolid treatment.

## Supplementary Material

dkae025_Supplementary_Data

## Supplementary data

Tables S1 to S4 are available as Supplementary data at *JAC* Online.
