# Correction to: Linezolid brain penetration in neurointensive care patients

**DOI:** 10.1093/jac/dkaf099

**Published:** 2025-03-19

This is a correction to: Arthur Hosmann, Miriam M Moser, Wisse van Os, Leon Gramms, Valentin al Jalali, Maria Sanz Codina, Walter Plöchl, Constantin Lier, Frieder Kees, Christoph Dorn, Karl Rössler, Andrea Reinprecht, Markus Zeitlinger, Linezolid brain penetration in neurointensive care patients, *Journal of Antimicrobial Chemotherapy*, Volume 79, Issue 3, March 2024, Pages 669–677, https://doi.org/10.1093/jac/dkae025

In the originally published version of the manuscript there was a typographical error in the last name of the fourth co-author. This should read “Leon Gramss”.

This error is outlined only in this notice to preserve the version of record.

