# Supplementary material for: Linezolid brain penetration in neurointensive care patients
Source: J Antimicrob Chemother. 2024 Feb 7;79(3):669–77. doi: 10.1093/jac/dkae025 (PMC10904716; doi:10.1093/jac/dkae025)
Supplement: dkae025_Supplementary_Data [file dkae025_supplementary_data.docx]

**Supplementary data**

|  | **demographics** | | | **laboratory investigations** | | | | | | | | **cerebrospinal fluid** | | | |
| --- | --- | --- | --- | --- | --- | --- | --- | --- | --- | --- | --- | --- | --- | --- | --- |
| **Patient** | **age [years]** | **sex** | **BMI** | **CRP [mg/dl]** | **leukocytes [G/L]** | **creatinine [mg/dl]** | **creatinine-clearance [ml/min]** | **y-GT [U/L]** | **GOT [U/L]** | **GPT [U/L]** | **albumin [g/L]** | **cell count (absolute/µl)** | **glucose (mg/dl)** | **lactate (mmol/L)** | **protein (mg/dl)** |
| 1 | 25 | m | 23.4 | 5.2 | 9.3 | 0.9 | 146.9 | 202 | 90 | 54 | 31.4 | 1196 | 103 | 5.1 | 29.2 |
| 2 | 53 | f | 29.4 | 32.0 | 27.4 | 1.7 | 47.5 | 531 | 190 | 108 | 27.1 | 10 | 86 | 4.4 | 111.7 |
| 3 | 48 | f | 29.1 | 1.7 | 7.2 | 0.3 | 341.3 | 461 | 37 | 95 | 30.9 | 21 | 57 | 2.1 | 35.3 |
| 4 | 55 | m | 26.2 | 10.9 | 6.0 | 0.9 | 107.9 | 662 | 331 | 211 | 28.0 | 188 | 59 | 5.3 | 102.2 |
| 5 | 61 | f | 23.8 | 2.7 | 13.8 | 0.6 | 99.5 | 374 | 48 | 39 | 26.1 | 794 | 48 | 6.3 | 235.6 |

**Table S1.** Demographics and laboratory investigations of each individual patient. BMI = body-mass index; CRP = C-reactive protein; f = female; m = male; GOT = glutamic oxaloacetic transaminase; GPT = glutamic pyruvic transaminases; y-GT = gamma-glutamyl transpeptidase

| **Patient** | **Matrix** | ***f*AUC_0-12h_ (mg⋅h/L)** | ***f*AUC_0-24h_ (mg⋅h/L)** | ***f*AUC_brain or CSF_/*f*AUC_plasma_** | **C_max_ (mg/L)** | **T_max_ (h)** | **t_1/2_ (h)** | **CL (L/h)** |
| --- | --- | --- | --- | --- | --- | --- | --- | --- |
| 1 | Plasma_free_ | 28.8 | 57.6 | NA | 9.6 | 1.0 | 1.9 | 20.8 |
| 1 | CSF | 22.6 | 45.2 | 0.8 | 4.7 | 1.0 | 2.4 | NA |
| 1 | Brain | 23.2 | 46.5 | 0.8 | 3.2 | 3.5 | 3.3 | NA |
| 2 | Plasma_free_ | 182.5 | 365.1 | NA | 24.8 | 1.0 | 9.9 | 3.3 |
| 2 | CSF | 153.1 | 306.1 | 0.8 | 16.2 | 2.0 | 10.6 | NA |
| 2 | Brain | 108.8 | 217.6 | 0.6 | 12.0 | 1.5 | 10.1 | NA |
| 3 | Plasma_free_ | 21.7 | 43.4 | NA | 9.9 | 1.0 | 2.5 | 27.7 |
| 3 | CSF | 21.7 | 43.5 | 1.0 | 4.9 | 2.0 | 3.0 | NA |
| 3 | Brain | 5.4 | 10.7 | 0.2 | 1.7 | 1.5 | 1.9 | NA |
| 4 | Plasma_free_ | 12.4 | 24.9 | NA | 6.2 | 1.0 | 2.0 | 48.2 |
| 4 | CSF | NA | NA | NA | NA | NA | NA | NA |
| 4 | Brain | 11.4 | 22.8 | 0.9 | 4.0 | 1.5 | 1.7 | NA |
| 5 | Plasma_free_ | 41.5 | 83.1 | NA | 13.1 | 1.0 | 2.9 | 14.4 |
| 5 | CSF | 41.5 | 82.9 | 1.0 | 8.0 | 2.0 | 3.0 | NA |
| 5 | Brain | 13.5 | 27.0 | 0.3 | 3.6 | 1.5 | 7.7 | NA |

**Table S2.** Pharmacokinetic parameters of linezolid for each patient in plasma, CSF, and cerebral interstitial fluid. *f*AUC = area under the concentration-time curve of free drug; C_max_ = maximum concentration; CL = clearance; CSF = cerebrospinal fluid; NA = not available; t_max_ = time to maximum concentration; t_½_ = terminal elimination half-life

|  |  | **MIC (mg/L)** | | | | | | | |
| --- | --- | --- | --- | --- | --- | --- | --- | --- | --- |
| **Patient** | **Matrix** | **0.125** | **0.25** | **0.5** | **1** | **2** | **4** | **8** | **16** |
| 1 | Plasma_free_ | 460.4 | 230.2 | 115.1 | 57.6 | 28.8 | 14.4 | 7.2 | 3.6 |
| 1 | CSF | 361.7 | 180.8 | 90.4 | 45.2 | 22.6 | 11.3 | 5.7 | 2.8 |
| 1 | Brain | 371.9 | 186.0 | 93.0 | 46.5 | 23.2 | 11.6 | 5.8 | 2.9 |
| 2 | Plasma_free_ | 2920.5 | 1460.2 | 730.1 | 365.1 | 182.5 | 91.3 | 45.6 | 22.8 |
| 2 | CSF | 2449.1 | 1224.5 | 612.3 | 306.1 | 153.1 | 76.5 | 38.3 | 19.1 |
| 2 | Brain | 1741.1 | 870.5 | 435.3 | 217.6 | 108.8 | 54.4 | 27.2 | 13.6 |
| 3 | Plasma_free_ | 347.1 | 173.6 | 86.8 | 43.4 | 21.7 | 10.8 | 5.4 | 2.7 |
| 3 | CSF | 347.9 | 174.0 | 87.0 | 43.5 | 21.7 | 10.9 | 5.4 | 2.7 |
| 3 | Brain | 85.9 | 42.9 | 21.5 | 10.7 | 5.4 | 2.7 | 1.3 | 0.7 |
| 4 | Plasma_free_ | 199.1 | 99.5 | 49.8 | 24.9 | 12.4 | 6.2 | 3.1 | 1.6 |
| 4 | CSF | NA | NA | NA | NA | NA | NA | NA | NA |
| 4 | Brain | 182.0 | 91.0 | 45.5 | 22.8 | 11.4 | 5.7 | 2.8 | 1.4 |
| 5 | Plasma_free_ | 664.7 | 332.4 | 166.2 | 83.1 | 41.5 | 20.8 | 10.4 | 5.2 |
| 5 | CSF | 663.2 | 331.6 | 165.8 | 82.9 | 41.5 | 20.7 | 10.4 | 5.2 |
| 5 | Brain | 215.9 | 107.9 | 54.0 | 27.0 | 13.5 | 6.7 | 3.4 | 1.7 |

**Table S3**. *f*AUC_0-24h_/MIC ratios of linezolid for each patient in plasma, CSF, and cerebral interstitial fluid. *f*AUC = area under the concentration-time curve of free drug; CSF = cerebrospinal fluid; MIC = minimal inhibitory concentration; NA = not available

|  |  | **MIC (mg/L)** | | | | | | | |
| --- | --- | --- | --- | --- | --- | --- | --- | --- | --- |
| **Patient** | **Matrix** | **0.125** | **0.25** | **0.5** | **1** | **2** | **4** | **8** | **16** |
| 1 | Plasma_free_ | 12.0 (100) | 10.9 (91) | 9.0 (75) | 7.0 (59) | 5.0 (41) | 2.7 (22) | 0.5 (4) | 0.0 (0) |
| 1 | CSF | 12.0 (100) | 12.0 (100) | 10.4 (87) | 8.0 (66) | 5.3 (44) | 0.5 (4) | 0.0 (0) | 0.0 (0) |
| 1 | Brain | 12.0 (100) | 12.0 (100) | 12.0 (100) | 10.4 (87) | 5.2 (44) | 0.0 (0) | 0.0 (0) | 0.0 (0) |
| 2 | Plasma_free_ | 12.0 (100) | 12.0 (100) | 12.0 (100) | 12.0 (100) | 12.0 (100) | 12.0 (100) | 12.0 (100) | 4.6 (38) |
| 2 | CSF | 12.0 (100) | 12.0 (100) | 12.0 (100) | 12.0 (100) | 12.0 (100) | 12.0 (100) | 11.9 (100) | 0.6 (5) |
| 2 | Brain | 12.0 (100) | 12.0 (100) | 12.0 (100) | 12.0 (100) | 12.0 (100) | 12.0 (100) | 7.9 (66) | 0.0 (0) |
| 3 | Plasma_free_ | 12.0 (100) | 9.6 (80) | 7.0 (58) | 5.1 (43) | 3.6 (30) | 1.8 (15) | 0.4 (4) | 0.0 (0) |
| 3 | CSF | 12.0 (100) | 12.0 (100) | 9.3 (78) | 6.6 (55) | 4.2 (35) | 1.8 (15) | 0.0 (0) | 0.0 (0) |
| 3 | Brain | 7.9 (66) | 6.0 (50) | 4.0 (34) | 1.9 (15) | 0.0 (0) | 0.0 (0) | 0.0 (0) | 0.0 (0) |
| 4 | Plasma_free_ | 7.9 (66) | 6.3 (52) | 5.0 (42) | 3.7 (31) | 2.2 (19) | 0.9 (7) | 0.0 (0) | 0.0 (0) |
| 4 | CSF | NA | NA | NA | NA | NA | NA | NA | NA |
| 4 | Brain | 8.8 (73) | 7.0 (59) | 5.5 (46) | 4.0 (33) | 2.3 (19) | 0.0 (0) | 0.0 (0) | 0.0 (0) |
| 5 | Plasma_free_ | 12.0 (100) | 12.0 (100) | 12.0 (100) | 9.0 (75) | 6.4 (53) | 3.8 (32) | 1.3 (11) | 0.0 (0) |
| 5 | CSF | 12.0 (100) | 12.0 (100) | 12.0 (100) | 10.6 (88) | 7.3 (61) | 4.4 (37) | 0.0 (0) | 0.0 (0) |
| 5 | Brain | 12.0 (100) | 12.0 (100) | 7.1 (59) | 4.9 (41) | 2.4 (20) | 0.0 (0) | 0.0 (0) | 0.0 (0) |

**Table S4.** *f*T>MIC values for each patient in plasma, CSF, and cerebral interstitial fluid. Data are presented in hours (% of dosing interval). MIC = minimal inhibitory concentration; *f*T>MIC = time during which concentrations exceed the MIC; CSF = cerebrospinal fluid; h = hours; NA = not available
